# Modeling and optimization of hydrogenation for crude oil by estimating hydrogen solubility in the solvent at different temperatures

**DOI:** 10.3389/fchem.2026.1876389

**Published:** 2026-08-11

**Authors:** Zhongtian Zhao

**Affiliations:** China National Aviation Fuel Group Corporation Limited, CNAF, Beijing, China

**Keywords:** harmony search algorithm, hydrogenation, hyper-parameter tuning, machine learning, modeling

## Abstract

A novel approach was introduced to predict the solubility of hydrogen in Athabasca bitumen sample by leveraging a hybrid approach based on Harmony Search Algorithm (HS) and AdaBoost. The solubility of H_2_ in the samples is of great importance for treatment of heavy hydrocarbon in petroleum processing and can help optimize processes. In the correlation of data, pressure and temperature were used as the inputs, while the hydrogen solubility was assigned the sole response for the modeling. This approach was applied to three popular regression models: K-Nearest Neighbors (KNN), Theil-Sen, and Lasso, resulting in hybrid models named HSA-KNN, HSA-TS, and HSA-LAS, respectively. The HS algorithm is used to optimize the hyperparameters of the Adaboost and base models, and then AdaBoost is applied to enhance the performance of the base models. The HSA-KNN model achieved an R^2^ score of 0.96466, MSE of 6.2790E-03, and a maximum error of 1.80485E-01, while the HSA-TS model achieved an R^2^ score of 0.96433, MSE of 6.3994E-03, and a maximum error of 1.40763E-01. The HSA-LAS model, on the other hand, achieved an R^2^ score of 0.89249.

## 1 Introduction

In petroleum industry, hydrogen is an important gas which is used for hydrogeneration process by which heavy hydrocarbons are treated to meet the standards by removing their impurities. It is basically described as liquid-phase chemical reaction where the separation takes place in liquid feed by reactions between hydrogen and the hydrocarbon components (Bilto and Uyar, 2026; Gao et al., 2026). For instance, to remove organosulfur compounds from liquid heavy hydrocarbons, hydrogen reacts with sulfur compounds to form gaseous products and treat hydrocarbons (Han et al., 2022; Morales-Valencia et al., 2021; Rodríguez Castillo et al., 2023). The modeling of hydrogenation in liquid phase is complex and the concentration of H_2_ in liquid phase is needed to be determined. The concentration of H_2_ in hydrocarbon is governed by its solubility in the feed, so solubility is one of the important parameters which should be accurately obtained for modeling of hydrogenation process in petroleum industry such as hydrodesulfurization process (HDS) for diesel treatment (Kalam et al., 2023; Safari and Vesali-Naseh, 2019; Wu et al., 2018). As such, for H_2_ related processes, knowing accurate values of H_2_ solubility at different temperatures and pressures is valuable which can help predict and optimize the process (Li et al., 2024).

The solubility of gases such as H_2_ in liquid can be modeled by various modeling approaches such as thermodynamics, molecular modeling, and data-driven models. Given that various components are available in heavy hydrocarbon as the liquid phase, the system is considered to be complicated, therefore, data-driven models such as machine learning can be used for obtaining and predicting hydrogen solubility in liquid hydrocarbons (Hadavimoghaddam et al., 2022). The experimental data is needed for data-driven models to be built, and also a proper method should be selected to be well suited for the dataset. The model can also investigate the changes in H_2_ solubility versus other input parameters such as temperature, pressure, etc. Methods of machine learning (ML) are versatile in process modeling and can be used for predicting hydrogen solubility in different hydrocarbons.

The realm of machine learning (ML) has become a focal point of interest in recent years owing to its broad applicability in diverse domains. ML employs statistical and computational techniques to enable machines to assimilate data, and make decisions or predictions based on that data. One of the main challenges in machine learning is to build accurate and reliable models that can generalize well to new data (Alpaydin, 2020; Lashari et al., 2021; Shinde and Shah, 2018).

The ensemble methods have become a potent tool for enhancing ML models’ effectiveness. Ensemble methods combine the predictions of multiple base models to obtain a final prediction that is often more accurate and robust than any individual model. AdaBoost is one such popular ensemble method that has been shown to be effective in improving the performance of several base models.

Another approach to improving the performance of machine learning models is tuning models through hyper-parameters optimization techniques. Harmony Search (HS) is a metaheuristic optimization algorithm that is inspired by the process of musical improvisation (Yang, 2009). HS has been utilized successfully in a variety of optimization problems, including feature selection, hyperparameter tuning, and model selection.

K-Nearest Neighbors (KNN) is a non-parametric method that has garnered substantial attention in the field of machine learning for its effectiveness in regression and classification tasks. KNN predicts the target value of a new sample by finding the k-nearest neighbors in the training set and using their average or weighted average as the prediction. KNN has been shown to perform well on many datasets, but its performance can be sensitive to the choice of the number of neighbors and the distance metric used (Cover, 1968; Cover and Hart, 1967).

For feature selection and regularization, Lasso Regression is a linear regression technique. Lasso adds an L1 penalty component to the usual least squares objective function, which favors sparsity and promotes the selection of only the most important characteristics. Lasso has been shown to be effective in handling high-dimensional datasets and reducing overfitting (Ranstam and Cook, 2018).

Theil-Sen Regression is a highly robust and effective linear regression method that surpasses the standard linear regression method in handling noisy datasets and outliers. This method is based on the median of pairwise slopes between data points, which provides a robust estimate of the underlying trend. The Theil-Sen Regression can be applied to datasets with high variability, as it is less affected by outliers than standard linear regression, thus making it highly reliable and accurate. Theil-Sen Regression has gained widespread attention and adoption in recent years owing to its capacity to handle challenging datasets that standard linear regression methods struggle with (Ohlson and Kim, 2015; Wilcox, 1998).

In this paper, for prediction of hydrogen solubility we propose a novel hybrid method that combines AdaBoost and HS optimization to improve the performance of three base models: K-Nearest Neighbors (KNN), Theil-Sen Regression (TS), and Lasso Regression (LAS). We refer to these hybrid models as HSA-KNN, HSA-TS, and HSA-LAS, respectively. These models are developed for hydrogen solubility in heavy hydrocarbon (bitumen) for the first time, and the results will be used for evaluation of solubility changes versus input features, i.e., *T* and *P*.

## 2 Problem statement

**FIGURE 1:**
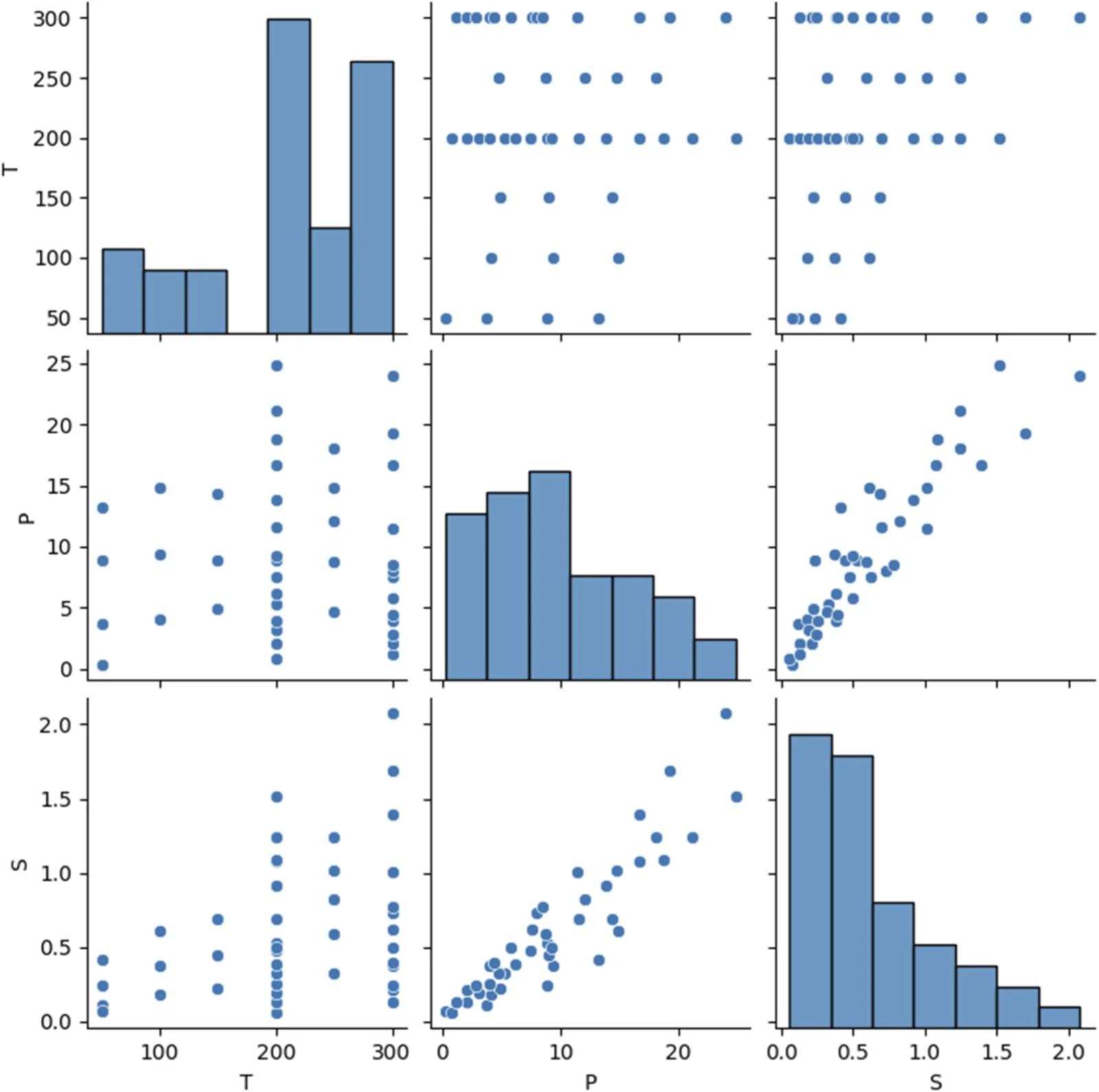
Pairwise scatter plot matrix of temperature (T), pressure (P), and hydrogen solubility (S). The off-diagonal plots show bivariate relationships between variables, while the diagonal plots represent the marginal distributions (histograms) of each variable.

The dataset used in this study contains the Solubility of hydrogen in Athabasca bitumen as reported in (Lal et al., 1999). Three parameters were taken into account including: temperature (T), pressure (P), and solubility (S), with a sole output which is S (Jin et al., 2023a). Other sources have reported the use of these data for machine learning analysis (Jin et al., 2023a; Jin et al., 2023b). The dataset contains 43 data points in total and are shown in Table 1 entirely. The solubility of hydrogen has been obtained after the thermodynamic equilibrium has reached in the system with the normal unit. Figure 1 presents a scatter matrix (pairplot) of the dataset variables, where the off-diagonal elements show pairwise relationships between temperature, pressure, and hydrogen solubility. The diagonal elements represent the marginal distribution of each variable in the form of histograms. These histograms share identical x- and y-axis scaling in each diagonal subplot because they depict frequency distributions of a single variable, rather than relationships between two different variables.

**TABLE 1:** The values of gas solubility in bitumen (Lal et al., 1999).

T (^°^C)	P (MPa)	S (g H_2_/kg solvent)
50	3.678	0.114
50	8.898	0.24
50	0.26	0.0737
50	13.16	0.414
100	4.064	0.178
100	9.331	0.375
100	14.84	0.615
150	4.899	0.225
150	8.932	0.447
150	14.352	0.689
200	0.783	0.057
200	2.024	0.128
200	3.127	0.193
200	3.955	0.259
200	5.334	0.33
200	6.127	0.386
200	7.506	0.478
200	8.884	0.526
200	9.195	0.498
200	11.607	0.694
200	13.883	0.918
200	16.71	1.081
200	18.779	1.09
200	21.158	1.243
200	24.845	1.517
250	4.695	0.321
250	8.713	0.59
250	12.087	0.82
250	14.794	1.014
250	18.104	1.244
300	1.187	0.131
300	2.024	0.217
300	2.852	0.241
300	3.954	0.38
300	4.402	0.397
300	5.815	0.494
300	7.539	0.62
300	7.953	0.729
300	8.469	0.777
300	11.47	1.008
300	16.71	1.392
300	19.295	1.693
300	24.018	2.075

## 3 Modeling of dataset

### 3.1 Base models

A ML algorithm that can be used for regression tasks is called K-Nearest Neighbors (KNN) regression. The algorithm is non-parametric, meaning it does not presuppose anything about the distribution of the data and only consults the training set when it is actually being used. KNN regression relies on the idea that similar data points tend to have similar outcomes (Bishop, 2006; Trevor et al., 2009). To perform the modeling in this study, the dataset was randomly partitioned into training (70%) and testing (30%). The split was performed at the sample level to ensure that both subsets preserve the overall distribution of temperature, pressure, and solubility values. The training set was used for model fitting and hyperparameter optimization, while the testing set was reserved exclusively for final performance evaluation and was not accessed during training or tuning. Moreover, external dataset was used to validate the robustness of model and prove the lack of overfitting.

Finding the K nearest neighbors in the training set allows the KNN algorithm to predict the output value for a new data point. Before an algorithm can be trained, a hyperparameter—the value of K—must be selected (Kramer et al., 2013). The distance metric used in KNN regression can be the Euclidean distance, Manhattan distance, or cosine distance, among others. Euclidean distance is the most common metric used in KNN, and it can be calculated as follows (Taunk et al., 2019):

$$d\left(x_{i},x_{j}\right)=\sqrt{\sum_{k=1}^{p}{\left(x_{ik}-x_{jk}\right)}^{2}}$$

Lasso regression, also referred to as L1 regularization, is a statistical method utilized in linear regression models to prevent overfitting. It achieves this by imposing a penalty on the magnitude of the coefficients, thus shrinking them towards zero (Tibshirani, 1996).

The Lasso regression technique aims to optimize the sum of squared differences between the predicted and actual values, while introducing a constraint on the sum of the absolute magnitudes of the regression coefficients. The constraint is imposed through the addition of a penalty term, which is proportional to the L1 norm of the coefficient vector (Sill et al., 2014):

Minimize:

$${RSS=\left\|Y-X\beta \right\|}^{2}$$

Subject to:

$${\left\|\beta \right\|}_{1}\leq t$$

where *Y* represents the vector of observed values, *X* stands for the design matrix, *β* denotes the vector of coefficients, *t* is the penalty parameter, and *||.||*_*1*_ denotes the L1 norm.

Lasso regression can be solved using various optimization algorithms, such as coordinate descent, least angle regression, or proximal gradient descent.

Theil-Sen regression is a nonparametric technique for estimating the slope of a linear relationship between two variables. The Theil-Sen regression algorithm works by first computing the slope among all possible pairs of data points and then taking the median of all the slopes (Ohlson and Kim, 2015; Wilcox, 1998). The median of the y-intercepts of the estimated intercept passing along the lines of the *x* and *y* values is then used to calculate the intercept. Mathematically, the Theil-Sen estimator for the slope can be written as (Ohlson and Kim, 2015):

$$\beta_{T}S=median\left[\left(yj-yi\right)/\left(xj-xi\right)\right]$$

where *i* and *j* are indices for any two distinct data points and median *[.]* denotes the median of a set of values (Rousseeuw and Croux, 1993).

### 3.2 Adaboost

Adaboost regression, or adaptive boosting regression, is a powerful ML algorithm that is useful for solving regression problems. It is based on the idea of boosting weak learners, which are models that have slightly better performance than random guessing. Adaboost regression combines these weak learners to form a strong learner that can make accurate predictions (Freund and Schapire, 1997; Schapire, 1990). Adaboost regression works by iteratively training a sequence of weak regression models on the data and then combining their predictions using a weighted sum. In each iteration, the algorithm assigns higher weights to the data points that were incorrectly predicted in the previous iteration, which causes the subsequent models to focus on the hardest examples (Solomatine and Shrestha, 2004). The algorithm can be summarized as follows (Bereta, 2019; Solomatine and Shrestha, 2004):

1. Initialize the weights of each data point to be equal.
2. For each iteration t = 1, 2, … T:
  - Develop a weak regression model *h*_*t*_ *(x)* on the data using the current weights.
  - Determine the errors on the training samples as:
$$\epsilon_{t}=\sum_{i=1}^{N}w_{i}^{\left(t\right)}\left|y_{i}-h_{t}\left(x_{i}\right)\right|$$
  - Determine the weight of the model as:
$$\alpha_{t}=\frac{1}{2}\ln\frac{1-\epsilon_{t}}{\epsilon_{t}}$$
  - Update the weights of the data points as:
$$w_{i}^{\left(t+1\right)}=\frac{w_{i}^{\left(t\right)}e^{-\alpha_{t}y_{i}h_{t}\left(x_{i}\right)}}{Z_{t}}$$
  - Where *Z*_*t*_ is a normalization factor that ensures that the weights sum to 1.
3. Combine the weak models by taking a weighted sum:

$$F\left(x\right)=\sum_{t=1}^{T}\alpha_{t}h_{t}\left(x\right)$$

The ultimate result of the algorithm is obtained as a weighted sum of the weak models, where the weights are assigned based on their efficacy in predicting the output values of the training data.

Adaboost regression has several advantages over other regression algorithms. Firstly, it is able to handle nonlinear relationships between the input variables and the target variable. Secondly, it is relatively insensitive to overfitting, which means that it can generalize well to new data. Finally, it is computationally efficient and can handle large datasets. In this study, AdaBoost serves as the ensemble wrapper around each base regression model, while hyperparameters are optimized using the Harmony Search algorithm.

### 3.3 Model construction framework

The proposed modeling framework follows a hybrid optimization–ensemble pipeline designed to enhance predictive accuracy and robustness of the employed models. First, the dataset consisting of temperature (T), pressure (P), and hydrogen solubility (S) is randomly divided into training (70%) and testing (30%) subsets. The training subset is used for model fitting and Harmony Search-based hyperparameter optimization, whereas the testing subset is strictly held out and used only for final model evaluation. Prior to model training, no explicit feature transformation is applied, ensuring consistency across all base models.

In the first stage, three base regression learners are defined: K-Nearest Neighbors, Theil–Sen regression, and Lasso regression. Each base learner is embedded within an AdaBoost regression framework to improve weak prediction performance through iterative reweighting of training samples.

In the second stage, the Harmony Search (HS) algorithm is employed as a global hyperparameter optimization strategy. HS tunes critical model parameters such as the number of estimators, learning rate, and base model-specific hyperparameters (e.g., number of neighbors for KNN and regularization strength for Lasso). The optimization objective is to minimize prediction error on validation data.

The final hybrid model structure is constructed by combining HS-optimized parameter selection with AdaBoost-based ensemble learning. This results in three hybrid models: HSA-KNN, HSA-TS, and HSA-LAS, where HS performs global search over the hyperparameter space and AdaBoost enhances model stability through weighted ensemble learning.

The overall workflow ensures sequential optimization (HS) followed by ensemble refinement (AdaBoost), enabling improved generalization performance for hydrogen solubility prediction by the combined methodology. No information from the testing set was used during model training or optimization, ensuring an unbiased evaluation of model performance. Finally, external data (unseen) was used to further validate the model performance and check the overfitting due to the small size of dataset for H_2_ solubility.

## 4 Results and discussions

**FIGURE 2:**
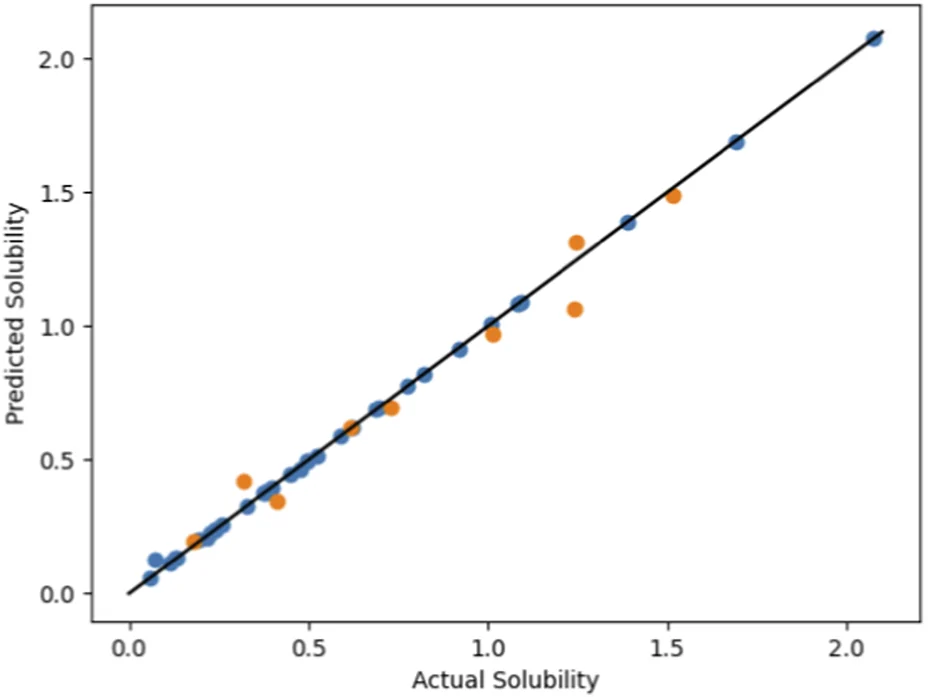
Actual (45-degree line) versus predicted values for HSA-KNN model. Blue points represent training data, and orange points represent validation data.

**FIGURE 3:**
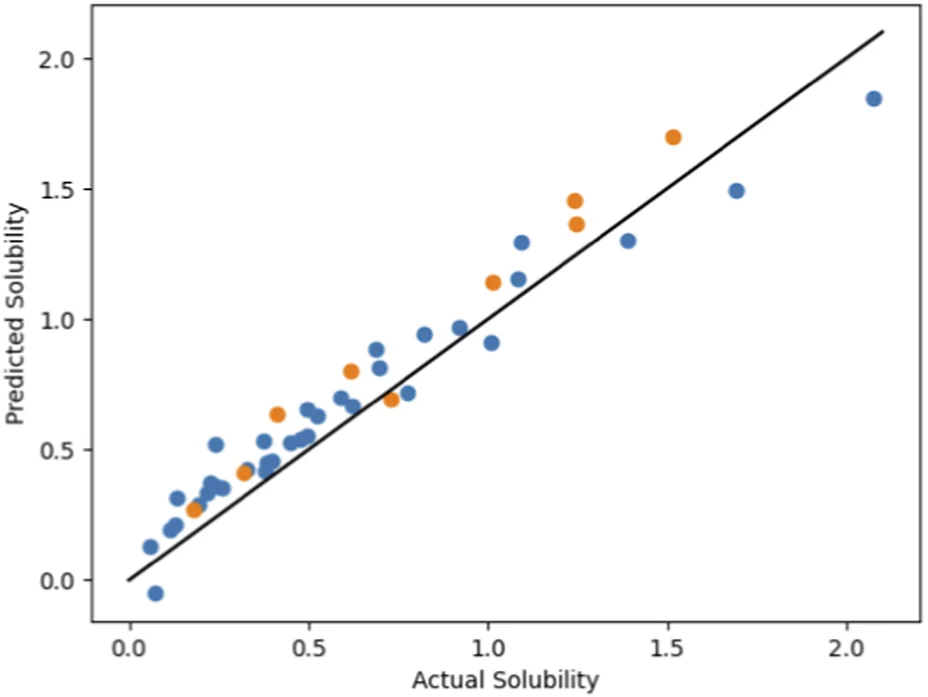
Actual (45-degree line) versus predicted values for HSA-LAS model. Blue points represent training data, and orange points represent validation data.

**FIGURE 4:**
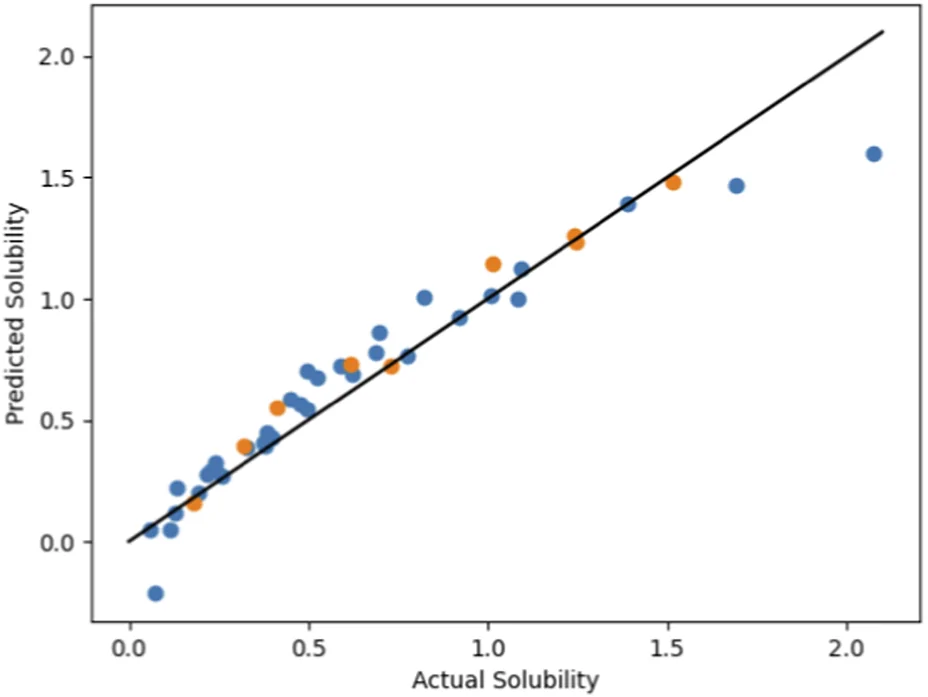
Actual (45-degree line) versus predicted values for HSA-TS model. Blue points represent training data, and orange points represent validation data.

Table 2 shows the hyperparameters and performance metrics of three hybrid models. This table clearly shows the higher accuracy of the HSA-KNN model compared to the other two models. In addition, Figures 2–4 also show the comparison of the values predicted by the models with the actual values, which confirm the same fact, and validity of the tuned models for solubility correlation. The blue points represent the training data predictions, while the orange points represent the validation (test) data predictions. Similar trend has been reported by Jin et al. (2023a), Jin et al. (2023b) for modeling H_2_ solubility data. Therefore, in the following, we use the HSA-KNN model as the main and superior model of this research to evaluate and analyze the process and solubility variations with the input parameters.

**TABLE 2:** Hyperparameters and performance metrics of the base models and hybrid models.

Model	Base model hyperparameters	Hybrid model hyperparameters	R^2^ score	MSE	Max error
HSA-KNN	algorithm = 'ball_tree’, n_neighbors = 4 weights = 'distance'	learning_rate = 0.91211 loss = 'square' n_estimators = 82	0.9646	6.279E-03	1.804E-01
HSA-TS	max_iter = 5 tol = 1.0706	learning_rate = 2.2227 loss = 'linear' n_estimators = 438	0.9643	6.399E-03	1.407E-01
HSA-LAS	alpha = 0.0355 tol = 0.007034	learning_rate = 0.7498 loss = 'square' n_estimators = 385	0.8924	2.344E-02	2.221E-01

**FIGURE 5:**
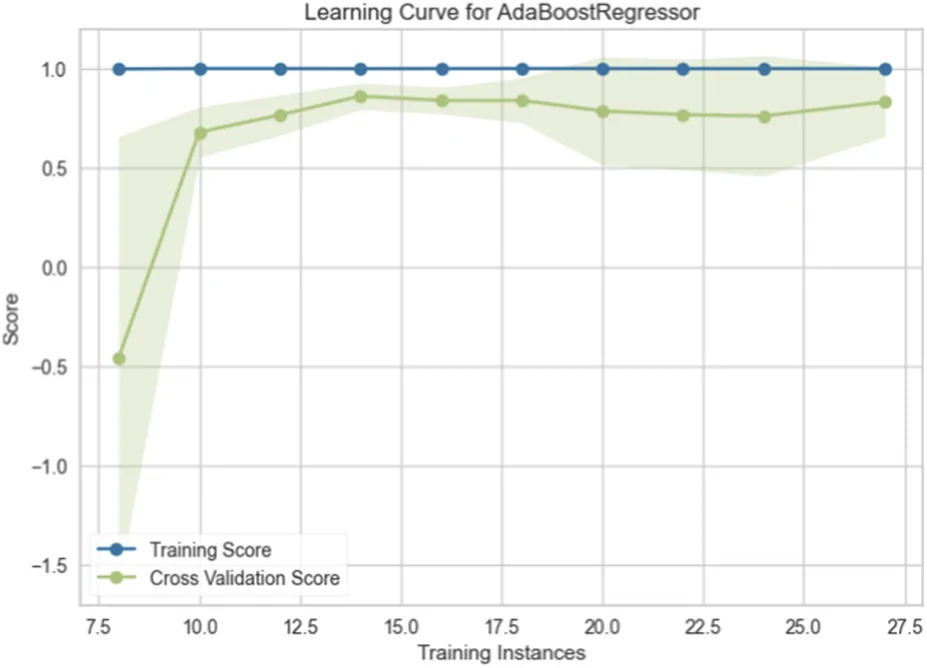
Learning curve for HSA-KNN model.

**FIGURE 6:**
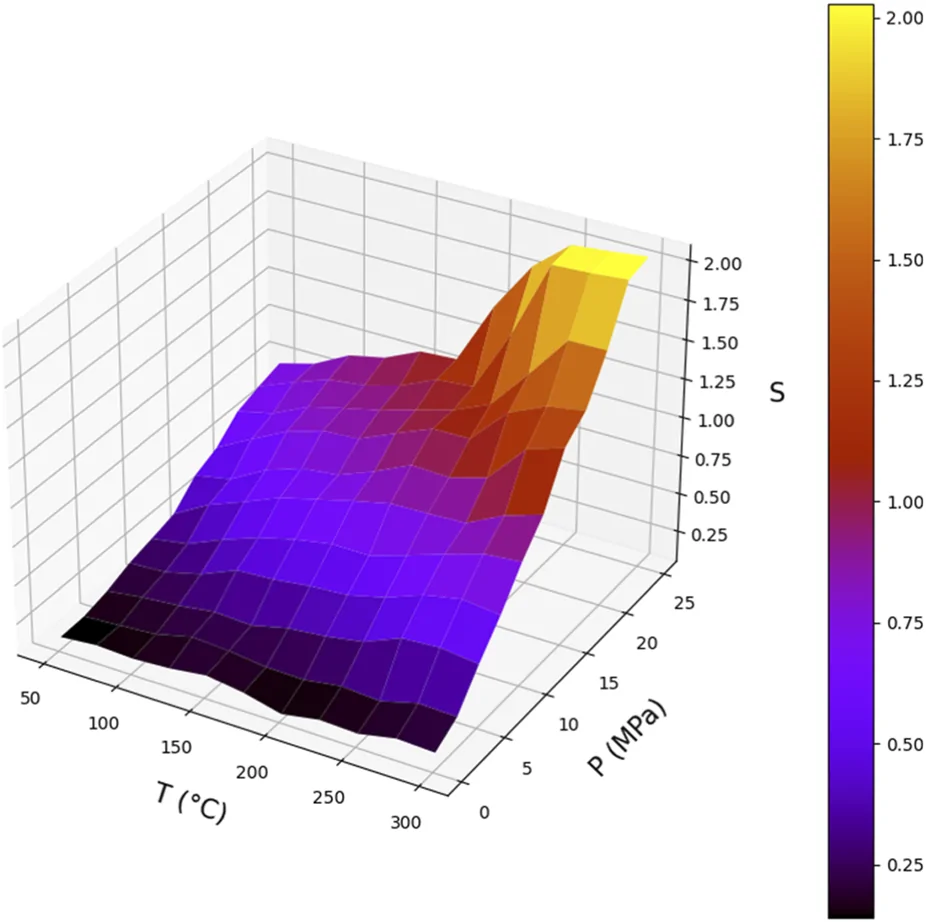
3D prediction surface using HSA-KNN model.

The Learning Curve of HSA-KNN is illustrated in Figure 5 in which the training and cross-validation scores is shown as a function of the training set size. Based on this figure this model is not over-fitted or under-fitted on the dataset of this study (Jin et al., 2023b). Figure 6 displays the 3D surface of the model, which depicts the relationship between the pressure and temperature inputs and the corresponding output values. The figure highlights that as the pressure and temperature inputs increase, there is an increase in the output value (Jin et al., 2023a; Jin et al., 2023b). The same figures for two additional models can be found in the appendix section (See Supplementary Appendix Figures A1, A2).

**FIGURE 7:**
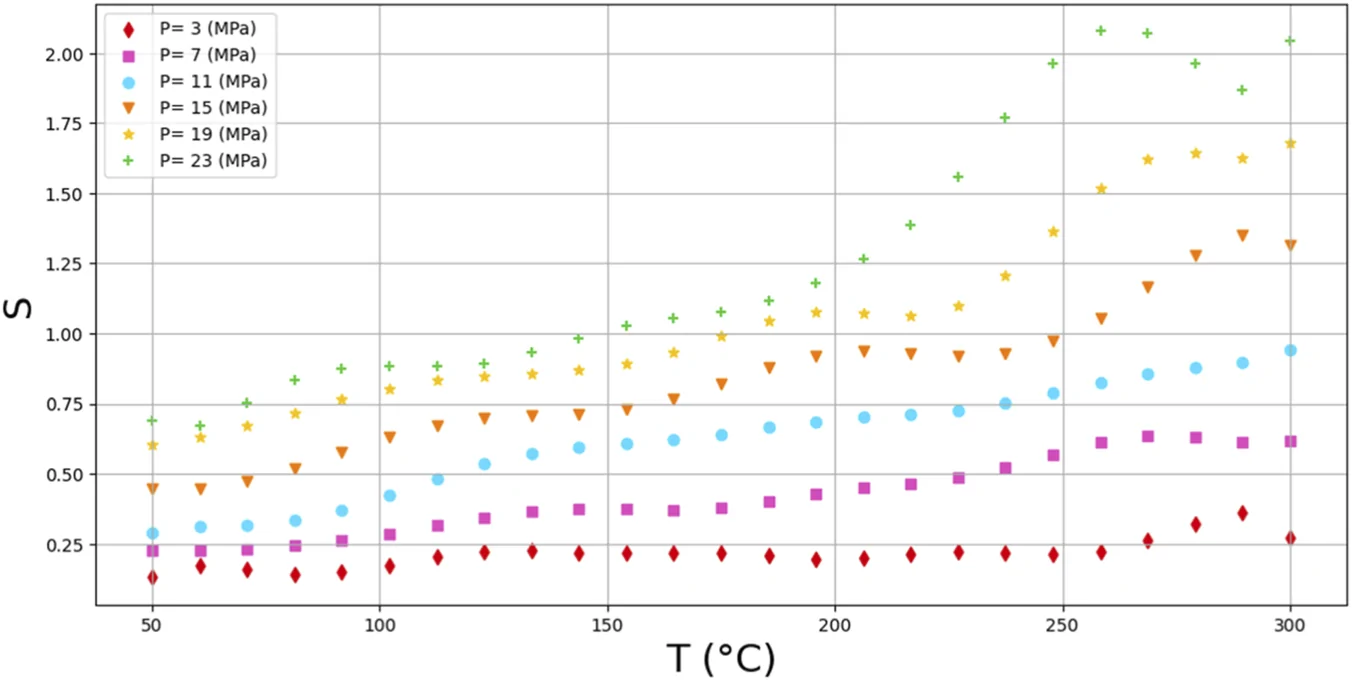
Individual Impact of Temperature on Solubility on multiple pressure levels.

**FIGURE 8:**
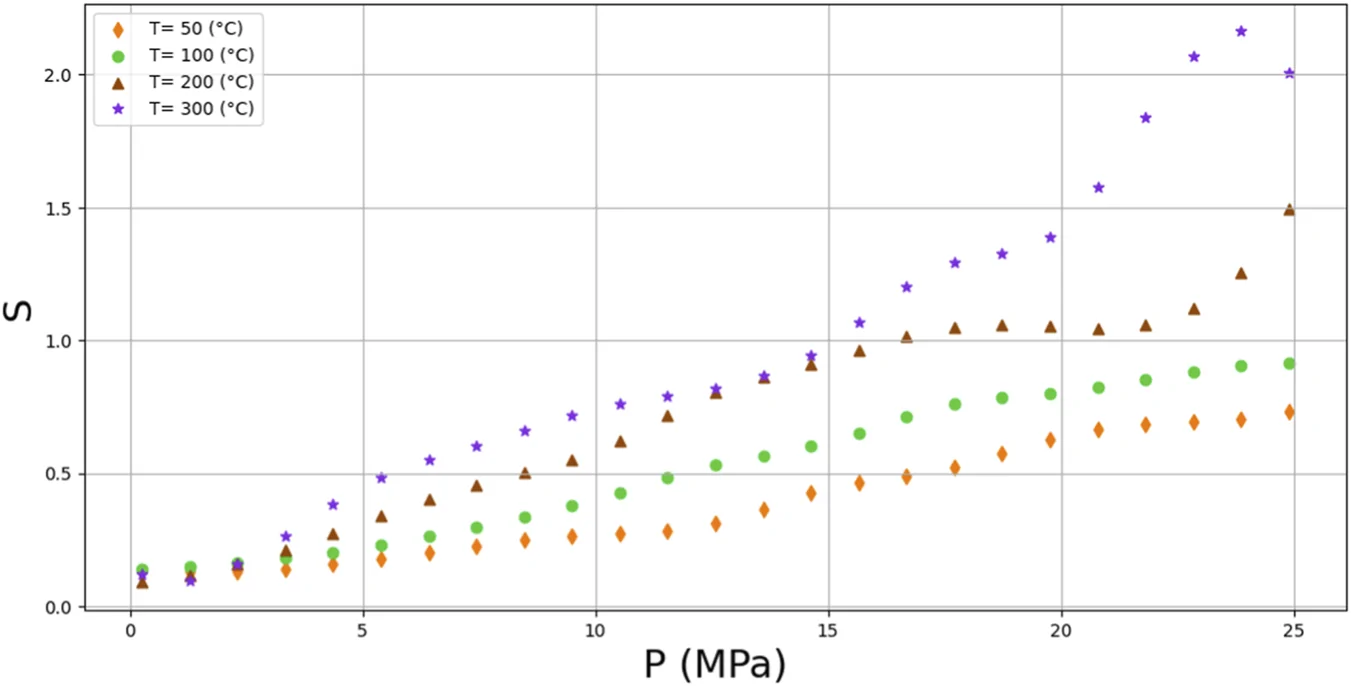
Individual Impact of Pressure on Solubility on multiple temperature levels.

The impact of both features on the output is further demonstrated in Figures 7, 8. The 2D figures are in agreement with the aforementioned observations derived from the 3D figure, indicating that an increase in both inputs leads to an increase in the output value (Jin et al., 2023a). The pressure enhancement can compress hydrogen and increases the solubility of hydrogen in the hydrocarbon sample which could be attributed to the enhanced intermolecular interactions between H_2_ and solvent molecules. Also, the temperature can enhance the reaction rate of H_2_ with the hydrocarbon sample which finally will increase the consumption of hydrogen and its mass transfer from gas towards the liquid phase. Also, the mass transfer rate and molecular diffusivity increase with rising temperature which leads to enhanced gas dissolution in the solvent phase (Li et al., 2024).

**FIGURE 9:**
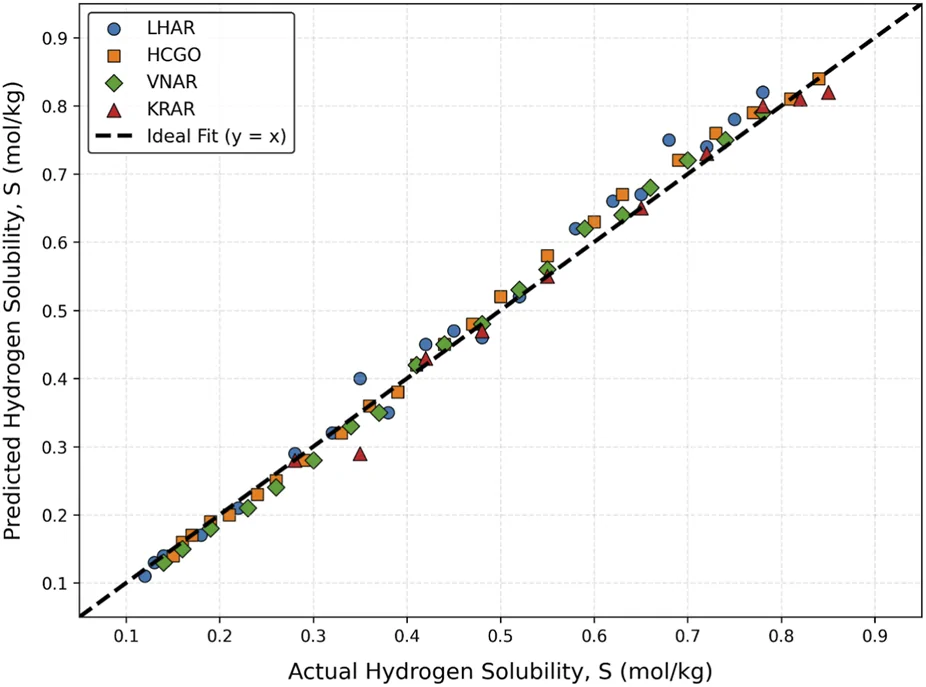
External validation of the optimized HSA-KNN model.

Finally, the optimized ML model is tested for estimation of H_2_ solubility for external dataset, and the results are illustrated in Figure 9 by comparing theoretical and measured data. The results are fitted using HSA-KNN model, which is the best model determined in this work to show the robustness and validity of the model for unseen dataset. The data have been collected from different sources for solubility of H_2_ in organic solvent (Ji et al., 2013). Given the small dataset size in this study, which is a limitation of current study, the external validation reveals great accuracy of the model, while no overfitting has been observed.

## 5 Conclusion

To summarize, this study has presented a novel approach that combines the Harmony Search Algorithm (HS) with AdaBoost to optimize and enhance the performance of three popular regression models: K-Nearest Neighbors (KNN), Theil-Sen, and Lasso. The models were optimized and applied for physical case study which is solubility of hydrogen in bitumen as the heavy hydrocarbon. The results demonstrate that the hybrid models, named HSA-KNN, HSA-TS, and HSA-LAS, outperform the base models in terms of accuracy, as evidenced by higher R^2^ scores, lower mean squared errors (MSE), and smaller maximum errors. The HSA-KNN model achieved the highest accuracy with an R^2^ score of 0.96466, MSE of 6.2790E-03, and maximum error of 1.80485E-01. The HSA-TS model achieved an R^2^ score of 0.96433, MSE of 6.3994E-03, and maximum error of 1.40763E-01. The HSA-LAS model achieved an R^2^ score of 0.89249, MSE of 2.3449E-02, and maximum error of 2.22157E-01. Both pressure and temperature were indicated to have direct relationship with the solubility of H_2_ in the hydrocarbon sample.

## Data Availability

The original contributions presented in the study are included in the article/Supplementary Material, further inquiries can be directed to the corresponding author.
