# Supplementary material for: Modeling and optimization of hydrogenation for crude oil by estimating hydrogen solubility in the solvent at different temperatures
Source: Front Chem. 2026 Aug 11;14:1876389. doi: 10.3389/fchem.2026.1876389 (PMC13503555; doi:10.3389/fchem.2026.1876389)
Supplement: Supplementary file 1 [file Supplementaryfile1.docx]

**Appendix**

**A. 3D representation of the solubility variations versus pressure and temperature**

Figure A1- 3D prediction surface of solubility simulated using HSA-LAS model.

Figure A2- 3D prediction surface of solubility simulated using HSA-TS model.
